# Is body image a predictor of women’s depression and anxiety in postmenopausal women?

**DOI:** 10.1186/s12888-020-02617-w

**Published:** 2020-05-06

**Authors:** Masoumeh Simbar, Soheila Nazarpour, Hamid Alavi Majd, Khadijeh Dodel Andarvar, Zahra Jafari Torkamani, Fatemeh Rahnemaie

**Affiliations:** 1 Midwifery and Reproductive Health Research Center, Shahid Beheshti University of Medical Sciences, Tehran, Iran; 2 Department of Midwifery and Reproductive Health, School of Nursing and Midwifery, Shahid Beheshti University of Medical Sciences, Tehran, Iran; 3 Department of Midwifery, Chalous Branch, Islamic Azad University, Chalous, Iran; 4 Department of Biostatistics, School of Paramedicine, Shahid Beheshti University of Medical Sciences, Tehran, Iran

**Keywords:** Menopause, Body image, Depression, Anxiety

## Abstract

**Background:**

Women in perimenopausal and postmenopausal period are at increased risk of depression and anxiety. Physiologic changes in menopause can change body’s appearance and function that may disturb body and then lead to anxiety and depression. This study aims to assess the relationship between body image with anxiety and depression among postmenopausal women.

**Methods:**

This descriptive study was performed on 307 women attending to the health centers in Tehran- Iran. Sampling was performed by a multi-staged randomized method. Data were collected by using Beck Depression questionnaire, Spielberger Anxiety Questionnaire, Fisher Body Image Questionnaire and Socio-demographic questionnaires. Data were analyzed by SPSS 21 and using *t*-test, Pearson and Spearman correlation tests and multiple linear regression methods.

**Results:**

The average age of the participants was 55.19 ± 4.034 years. Mean scores for body image, anxiety and depression were 163.26 ± 20.38, 12.00 ± 7.71 and 42.70 ± 8.40 respectively. Fifty five percent of women had mild to severe depression and 83.7% of them had mild to severe anxiety. Total score and all domains of body image had a negative correlation with depression and anxiety scores (*P* < 0.001). Multiple linear correlation showed that body image is a predictor for depression and anxiety (*P* < 0.001).

**Conclusions:**

Body image of women can be effective on occurrence of depression and anxiety in menopause. Therefore, women’s health policies should consider body image to control cognitive problems including depression and anxiety in menopause.

## Background

Menopause is defined as the reproductive condition after 12 consecutive months of amenorrhea, as a result of the complex hormonal changes that accompany the reduction in ovarian follicles [1, 2]. It is a critical period in women’s life which is associated with significant physical and mental changes [3, 4]. In menopause, decreasing of estrogen causes several complications including vasomotor symptoms, urogenital atrophy, osteoporosis, cardiovascular diseases, breast and skin atrophy, cancer, decreasing cognitive function and increasing sexual problems [5, 6].

For majority of women, menopause is an important event in life that impacts on their mental health [7]. Hormonal fluctuations in postmenopausal period are associated with psychological symptoms such as depressive symptoms and disorders which are common among midlife women [8]. Several studies attempted to clarify the role of menopausal status on developing depression and anxiety in midlife period. However, there is a controversy about causal effects of menopause on depression and anxiety. Besides, anxiety has been less studied, in spite of its abundance incidence in midlife women and its effects on women’s function [9].

Women in perimenopausal and postmenopausal period are at increased risk of depression and anxiety [9]. Increased clinical and subclinical risks of depression are observable in low estrogenic status such as postmenopausal, however, menopause does not directly cause depression and neurotransmitters thought to be most *directly* associated with *depression* [10]. Besides, Women with menopausal symptoms such as hot flushes, night sweats, vaginal dryness and dyspareunia are more likely to report anxiety and/or depressive symptoms. Bothersome vasomotor symptoms could also be associated with sleep disturbances, which in turn can increase reports of anxiety and depressive symptoms [8]. Consequently, depression and cognitive disorders can increase load of disease among women in elderly [11].

Besides, while many women show depression and anxiety in menopause, the reasons for these disorders are not attributable only to menopause. The effects of socioeconomic factors, such as educational level and ethnicity [8] as well as psychosocial factors such as life styles, culture, interpersonal relations and body image cannot be ignored [12].

Furthermore, midlife women are in the process of aging and so experience elderly related anxiety. It is said that aging related physical changes may lead to a negative body experience and so anxiety [13, 14].

During menopause, decreased estrogen levels lead to an increase in fat mass, and a relative increase in the androgen / estrogen ratio changes the body’s fat distribution pattern. So that more fat is stored in the abdominal area and leads to abdominal fat distribution (type of Android obesity) [15]. In addition to hormonal changes, other factors such as lifestyle and unhealthy behavioral factors during this period, such as lack of exercise and increased consumption of unhealthy foods can also affect these changes [16]. Increased fat mass and changes in its distribution pattern could negatively affect the body image of middle-aged women [17].

Besides, The symptoms of aging such as gaining weight, changing shape, wrinkles, losing muscle mass, changes in skin, hair and sexual function, flushing and osteoporosis can alter women’s perceptions and feeling about their own body [3, 18–20]. Menopausal physical symptoms may lead to perception of losing attractiveness, negative body image, decreasing self-esteem and mental vitality so impact on women’s mental health [21].

Recent evidence has shown that as few as 12% of older women are satisfied with their body size [22]. Thus, there is an equivocal relationship between body image and menopausal mental health [21].

To date, studies on body image have focused on younger women, as body image issues have usually been associated with this group [22]. Most recent studies have been concentrated on adolescents’ and adults’ body image, especially related to eating disorders, and there are a few studies about body image in midlife women or in menopausal transition [4, 23]. However, recent evidence has suggested that aging women are expressing similar concerns. Some studies shown that women are anxious about aging, especially in terms of their appearance and weight gain [24, 25].

Similar to puberty and pregnancy, the menopausal transition is a milestone in a woman’s life, with accompanying bodily changes and symptoms that can greatly affect her body image [18]. Therefore, attention to menopause and the change in body image in postmenopausal women can be just as important as in adolescents. So that, current studies showed that body dissatisfaction may be a problem in midlife women and needs more attention [26].

It has been shown that in addition to similar effective factor on young women’s body satisfaction, midlife women are facing with other important factors including menopausal- and aging-related anxiety and depressive symptoms [14]. Therefore, this study aims to assess relationship between body image with depression and anxiety among postmenopausal women.

## Method

### Study design

This was a descriptive correlational study on 307 postmenopausal women attending to the health centers in Tehran-Iran. Sampling was performed using multistage sampling method. Inclusion criteria were: natural menopause, no surgical or early menopause (earlier than 40 years old), no history of chronic physical and mental diseases, and no history of psychological disease, no acute stress in the previous months and no use of herbal medicines containing phytoestrogens or supplements of sexual hormones.

### Sampling method

Sampling was performed by a multi-stage sampling method. In the first stage four district of Tehran from north, south, east and west were selected. In the second randomized cluster sampling stage, 8 centers were randomized and then in the third stage quota sampling was performed. Written informed consent was obtained from all participants. Then they completed the questionnaires of the study.

### Tools for data collection

Tools for data collection were 4 questionnaires including; 1) Beck depression questionnaire, 2) Spielberger anxiety questionnaire, 3) Fisher body image questionnaire, and 4) a researcher made demographic questionnaire.

#### Beck depression questionnaire

The second version of Beck depression questionnaire (inventory II) (BDI-II) is a popular and self-completion tool for screening of depression among above 13 years old individuals. This questionnaire has two versions with 21 items and also with 13 items. It has the ability to assess severity and type of depression [27–29]. In the present study the Persian version of the questionnaire with 21 items was used [27, 28, 30]. All 21 items are measured with 4 level scales, scoring from 0 to 3. These items are about sadness, pessimism, past failure, loss of pleasure, guilty feeling, punishment feeling, self-dislike, self-criticalness, suicidal thoughts or wishes, crying, agitation, loss of interest indecisiveness worthlessness, loss of energy, changes in sleeping pattern, irritability, changes in appetite, concentration, difficulty tiredness or fatigue and loss of interest in sex. Therefore, this questionnaire measures the different degrees of depression from mild to very severe and scored from 0 to 63 [31]. Several studies assessed and confirmed content validity, construct validity and reliability as well as the cut of point of the questionnaire [27–30].

Validity and reliability of Persian version of the questionnaire was assessed by Taheri Tanjani and colleagues among elderly population. Intra-cluster correlation (ICC) was calculated 0.81. Internal stability was obtained by calculating α-Cronbach and half splitting 0.93 and 0.64, respectively. Convergent validity of the Beck questionnaire were also assessed by calculating of correlation with GHQ-28 and its four constructs and the total correlation was obtained 0.8 [32].

#### Speilberger anxiety questionnaire

This questionnaire was developed by Spielberger and includes 20 questions with 4 choices scoring 1 to 4. The range of scores is from 20 to 80. Scores from 35 to 45, 46–56 and ≥ 57 are categorized from low, middle to sever anxiety [33]. Validity and reliability of Spielberger questionnaire was assessed and confirmed in the previous studies [34]. Validity and reliability of the Persian version of the Speilberger questionnaire was also assessed and confirmed in a few studies [35–38]. Mahram (1993) reported construct validity 0.99 and 0.95 and α-Cronbach’s 0.91 and 0.9, for State and trait anxiety questionnaires, respectively [36]. Kanani and colleagues reported reliability 0.9 and 0.89 for state and trait anxiety questionnaires [35].

#### Fisher body image questionnaire

It was developed by Fisher and has 46 items [39]. Each item is responded by 5 level choices from 1 to 5 to very unsatisfied to very satisfied. The range of total scores is from 46 to 230 and high scores are showing positive body image. The domains includes: head and face with 12 items, upper limbs with 10 items, lower limbs with 6 items and 18 items related to attitudes about characteristics of whole body [40]. Validity of the test was assessed in Iran [40, 41]. Test-retest Pearson correlation coefficient 0.84 was calculated in the previous studies [40, 41]. In a study by Nazarpour and Khazai (2012), reliability of the Fisher body Image questionnaire was calculated by α-Cronbach, Spearman Brown, Guttman Split-half coefficient of 0.918, 0.861 and 0.861 respectively [42].

The scores of all three above mentioned questionnaire were also calculated to percent by using the following formula: (Score-Min) /(Max-Min) × 100.

#### The socio-demographic questionnaire

The socio-demographic questionnaire includes 10 questions about age, age of menopause, number of children, educational level of the women and her husband, women education and husband education, housing situation and adequacy of family’s monthly income.

### Statistical analysis

The data were analyzed using SPSS-21. The correlations between variables were calculated by Pearson test for normal distributed continuous variables and Spearman test for ordinal values and non-normal distributed variables. Also, linear multiple regression was used for determining predictive variables for depression and anxiety. For linear multiple regression, depression was considered as the dependent variable. Once again, anxiety was considered as the dependent variable.

The assumptions for the multiple linear regression models were that depression and anxiety are related to scores of Body Image. First, in multiple linear regressions, depression score was considered as dependent variables, and score of total body image were the main variable whose relation to the depression score was evaluated.

Second, in multiple linear regressions, anxiety score was considered as dependent variables, and score of total body image were the main variable whose relation to the anxiety score was evaluated. Age, duration of menopausal period, marital status, job of women, educational level of women and her spouse, housing situation, the adequacy of monthly household income were included in regression models as they were considered potential confounders, as they have been shown by other studies to be related to the depression and anxiety in menopause.

The interactions between confounders were assessed. However, these interaction terms were not included in the final model as they were not statistically significant.

The level of significance was set at *p* less than 0.05.

## Results

Three hundred and seven women participated in the study. The eligibility rate among the postmenopausal women who agreed to be participated, and the participation rate among those found to be eligible were 85% and 98%, respectively. The reason for not participating in the study may be due to menopausal complications.

The average age of the participants and average duration of menopause were 55.19 ± 4.03 (mean ± SD) years and 6.10 ± 4.062 years, respectively. Ninety six percent (295 women) were married and 77.5% (238 women) were housewife. The participants’ demographic characteristics are presented in the Table 1.

**Table 1:** Demographic characteristics of the postmenopausal women (*N* = 307)

Variables		Mean ± SD/n(%)
Age (years)		55.19 ± 4.034
Duration of menopause^a^ (months)		6.10 ± 4.602
Number of children		3.22 ± 1.601
Marital Status	Married	295 (96.1)
	Single/ Widow/ Divorced	12 (3.9)
Occupation	Housewife or retired	272 (88.6)
	Employed	35 (11.4)
Education	Illiterate	48 (15.6)
	Diploma and under diploma	211 (68.7)
	Higher diploma	48 (15.6)
Job of Husband	Employed	163 (53.1)
	Retired or unemployed	134 (43.6)
Education of husband	Illiterate	32 (10.4)
	Diploma and under diploma	222 (72.3)
	Higher diploma	53(17.3)
Housing situation	The owner	238 (77.5)
	Non-owner	69 (22.5)
The adequacy of monthly household income	Less than adequate	175 (57.0)
	Adequately or with saving	132 (43.0)

^a^ Menopause is defined as the time when there have been no menstrual periods for 12 consecutive months

Mean score for body image was 163.26 ± 20.80 (63.73 ± 11.31%). The satisfaction with “face & head” (67.38 ± 10.91%) and “lower limbs” (58.06 ± 17.57%) obtained the highest and lowest scores, respectively.

Mean score for depression was 12.00 ± 7.71 (19.05 ± 12.24%). Results showed that 55% (169) of women are affected by mild to severe depression. Mean score for anxiety was 42.70 ± 8.40 (37.83 ± 14.00%). Finding demonstrated that 83.7% (257) of women are affected by mild to severe anxiety. The scores of depression and anxiety are presented in the Table 2.

**Table 2:** Frequency of different levels of depression and anxiety among postmenopausal women and the mean scores of women’s depression and anxiety

Mental Health Condition		
Depression	N	%
Normal (1–10)	138	45.0
Mild Mood (11–16)	83	27.0
Borderline depression (17–20)	43	14.0
Moderate depression (21–30)	39	12.7
Severe depression (31–40)	3	1.0
Extreme depression (> 40)	1	0.3
Total score (Mean ± SD)	12.00 ± 7.71	19.05 ± 12.24 (0–100)
Anxiety	N	%
Normal (20–34)	50	16.3
Mild (35–45)	145	47.2
Moderate (46–56)	100	32.6
Severe (≥57)	12	3.9
Total score (Mean ± SD)	42.70 ± 8.40	37.83 ± 14.00 (0–100)

Pearson correlation coefficients demonstrated significant negative correlations between scores of body image and its dimensions with depression and all its dimensions; as well as significant negative correlations between scores of body image and its dimensions with anxiety and all its dimensions (*P* < 0.001) (Table 3).

**Table 3:** The correlation between Depression and Anxiety with Body Image and the Demographic factors

Demographic factors	Depression	Anxiety
Body Image		
Head & face	*r*_*p*_ = − 0.353^*^	*r*_*p*_ = − 0.333^*^
Upper limbs	*r*_*p*_ = − 0.505^*^	*r*_*p*_ = − 0.347^*^
Lower limbs	*r*_*p*_ = − 0.437^*^	*r*_*p*_ = − 0.318^*^
Overall	*r*_*p*_ = − 0.525^*^	*r*_*p*_ = − 0.394^*^
Total Body Image	*r*_*p*_ = − 0.532^*^	*r*_*p*_ = − 0.406^*^
Age	*r*_*s*_ = − 0.138^*^	*r*_*s*_ = − 0.112
Duration of menopause	*r*_*s*_ = − 0.073	*r*_*s*_ = − 0.034
Number of children	*r*_*s*_ = 0.080	*r*_*s*_ = 0.112
Education	*r*_*s*_ = −0.303^***^	*r*_*s*_ = − 0.187^**^
Education of husband	*r*_*s*_ = − 0.343^***^	*r*_*s*_ = − 0.223^***^
The adequacy of monthly household income	*r*_*s*_ = − 0.401^***^	*r*_*s*_ = − 0.342^***^
Marital Status	*t =* 2.308^*^	*t =* 890
Occupation	*t =* 3.363^**^	*t =* −0.309
Job of Husband	*t =* 0.100	*t =* 0.954
Housing situation	*t =* −2.994^**^	*t =* −4.870^***^

*P < .05. **P < .01. ***P < .001. Test: *r*_*p:*_ Pearson correlation coefficient; *r*_*s*_: Spearman’s correlation coefficient **(**Since the data related to age, duration of menopause, and number of children was shown to have non normal distribution, Spearmen correlation was used for data analysis. Besides, educational level and monthly adequacy of family’s income were ordinal variables); *t*: Independent sample t-test

The results demonstrated a negative correlation between depression and the age of women. There were also negative correlations between educational level of women and the educational level of husbands and also monthly adequacy of family’s income with the scores of depression and anxiety (Table 3).

Besides, *t*-test showed higher depression scores among married women who are living with their husbands comparing with widows and single women (*P* = 0.022). Furthermore, employed women obtained significant lower scores of depression comparing with housewife or retired women (*P* = 0.001). Moreover, women who were owner of housing had significant lower scores of depression (*P* = 0.003) and anxiety comparing with non-owner ones. (*P* < 0.001).

The multiple linear regression model for depression showed that body image is a predictor for depression, whereas with increase 1 unit in body image, depression decreases 0.161 unit (*P* < 0.001). Also, age of women (*P* = 0.011) and adequacy of monthly family’s income (*P* = 0.008) were also predictive of depression, while depression decrease with increasing age and adequacy of the family’s monthly income (Table 4).

**Table 4:** Predictors of Depression and Anxiety

Dependent variables	Predictors	Multiple linear regression		
		*P* value	Beta	B
Depression	Body Image	< 0.001	−0.433	−0.161
	Age	0.011	−0.146	−0.279
	The adequacy of monthly household income^a^	0.008	−0.149	−1.022
Anxiety	Body Image	< 0.001	−0.325	−0.131
	Occupation^b^	0.017	0.134	3.547
	Housing situation^c^	0.002	0.161	3.240
	The adequacy of monthly household income^a^	0.003	−0.180	−1.344

^**a**^Classification of the adequacy of monthly household income: 0 (Zero). Less than adequate, 1. Adequately or with saving; ^**b**^Classification of Occupation: 1. Housewife or retired, 2. Employed; ^**c**^Classification of Housing situation: 1. Owner, 2. Non-owner

Also, the multiple linear regression models for anxiety demonstrated that body image is a predictor for anxiety, whereas with a unit increase of body image, anxiety declines 0.131 unit (*P* < 0.001). The results also showed that the employment status (*P* = 0.017), housing situation (*P* = 0.002), adequacy of monthly family’s income (*P* = 0.003) are from predictors of anxiety, whereas increasing the income, ownership of housing and employment of the women predicts decreasing anxiety (Table 4).

## Discussion

This study showed that body image is associated and statistically predictor for depression and anxiety with a logistic regression model adjusted for age, duration of menopausal period, marital status, job of women, educational level of women and her spouse, housing situation, the adequacy of monthly household income for the first time in Iranian postmenopausal women.

The results demonstrated a negative correlation between women’s depression with their body image in menopause. These results are in consistent with finding of other studies [18, 23, 43]. A cross sectional study on African American and Caucasian midlife women aged 42 to 52 years old women showed middle aged women with poor body image and perceived unattractiveness experience depression nearly twice more than women who are dissatisfied with their body [23]. A path analysis also showed a negative correlation between body image and depression in perimenopausal Korean women [43]. Turkish women with lower scores of depression had higher scores of body image [18]. These finding demonstrated that nationality could not moderate the associations between the body image and depression. Jackson et al. (2014) also showed that race did not significantly moderate the associations between the body image variables and clinically significant levels of depressive symptoms [23].

Besides, the studies about the relationship between body image and depression in other groups or genders showed similar results. A study on 11,782 adult individuals in Korea showed a correlation between body image and depression. They showed that the risk of depression increase among individuals with the perception of their own obesity or thinness [44]. A systemic review also demonstrated the relationship between depression with self-body image [45]. Kim and colleagues (2018) in a study on 3318 adults found that among women with obesity, those who under-perceived their weight status reported fewer depressive symptoms compared to those who accurately perceived their weight status. Besides, among women with normal weight, those who over-perceived their weight status reported more depressive symptoms compared to those who accurately perceived their weight status. They concluded that awareness-oriented strategies for obesity prevention and weight management focused on providing information on weight status may need to consider unintended consequences of accurate weight perception on mental health among individuals with obesity, particularly among women [7].

Thus, the present study also suggests interventions to improve real body image of menopausal women to control weight and prevent mental conditions.

Finding also showed a negative relationship between body image and anxiety. The studies are limited about the relationship between body image with anxiety among perimenopausal women. A study shows association between body dissatisfaction, importance of appearance, aging anxiety and depression [46]. Another study on adolescents showed a significant relationship between body image with anxiety [47].

Besides, the relationship between body image with depression and anxiety were also studied in different patients. For instance, different studies showed significant correlation between body image with depression or anxiety or both, in women affected by breast cancer [48], among women affected by poly cystic ovary syndrome [49], also in women with the Autosomal Dominant Polycystic Kidney Disease (ADPKD) [50] and individuals with history of head and neck surgery [51], or patients with oral cavity cancer [52]. Therefore, it seems negative perception and dissatisfaction with body appearance and its function is a main concern that lead to mental conditions including depression and anxiety [53–55].

In the present century that societies define unreal definitions for young women’s appearance, physiologic changes during menopause may be in oppose with women’s ideals for body fitness. Consequently, many women express anxiety about ageing and its effect on appearance [56, 57]. Studies have also highlighted how anti-aging discourses are promoting unrealistic body norms, which have shown to contribute to poor body image and altered health behaviors [22]. In addition, it seems many women perceived physiologic changes of menopause as the initiation of aging. Therefore, health care policy makers and planers should consider women’s perception about their own body changes to prevent mental condition such as anxiety and depression to improve women’s mental health during menopausal period of life [21].

Women experience several psychosocial complications, special feeling, attitude and concerns in addition to physical changes due to hormonal changes [58]. The most common attitude that women experience during menopausal period is losing attractiveness and fitness [59]. Although, negative body image cannot be the only risk factor for depression and anxiety in postmenopausal women and many other factors such as occurrence of stressful events of life for instance leaving children, empty nest syndrome, or incidence of diseases and worries about sexual dysfunction and femininity roles are from contributing factors [60, 61] (Azizi et al. 2019). However, body image is a significant associated factor for depression and anxiety during menopause.

This study could not show the causes and effect relationship between body image with depression and anxiety. Therefore, longitudinal studies are suggested to show cause and effects roles of these variables. Besides, women’s empowerment and copying strategies such as copying with the symptoms of menopause, menopausal women empowerment, health education, interventions and using complementary and alternative medicines to provide relief from menopausal symptoms are recommended [62].

The present study measured only the correlation between body image and depression. While, other studies measured also the relationship between depressions with BMI, perceived attractiveness, body discordance [23] and weigh satisfaction [23, 44]. It seems individuals’ BMI, and their perception about weight and attractiveness construct their body image and satisfaction [63].

## Conclusion

Body image is associated with, and a statistically predictor for depression and anxiety. Therefore, special attention to improve women’s body image in menopause is essential to improve women’s mental health in menopause. The main point that should be considered in health programs is planning of programs to improve women’s acceptance of changes in menopause as a physiologic process and a positive and correct perception about self-body image to promote women mental health during postmenopausal period of life.

## Data Availability

The datasets used and/or analysed during the current study are available from the corresponding author on reasonable request.

## Abbreviations

BDI-II: Beck depression questionnaire (inventory II)
GHQ-28: General Health Questionnaire-28
ICC: Intra-class correlation
ADPKD: Autosomal Dominant Polycystic Kidney Disease
SPSS 21: Statistical Package for the Social Sciences (version 21)
