# Correction to: Is body image a predictor of women’s depression and anxiety in postmenopausal women?

**DOI:** 10.1186/s12888-020-02685-y

**Published:** 2020-06-04

**Authors:** Masoumeh Simbar, Soheila Nazarpour, Hamid Alavi Majd, Khadijeh Dodel Andarvar, Zahra Jafari Torkamani, Fatemeh Alsadat Rahnemaei

**Affiliations:** 1 Midwifery and Reproductive Health Research Center, Shahid Beheshti University of Medical Sciences, Tehran, Iran; 2 Department of Midwifery and Reproductive Health, School of Nursing and Midwifery, Shahid Beheshti University of Medical Sciences, Tehran, Iran; 3 Department of Midwifery, Chalous Branch, Islamic Azad University, Chalous, Iran; 4 Department of Biostatistics, School of Paramedicine, Shahid Beheshti University of Medical Sciences, Tehran, Iran

**Correction to: BMC Psychiatry (2020) 20:202.**

**https://doi.org/10.1186/s12888-020-02617-w**

Following publication of the original article [1], the authors identified a mistake in the last author’s name.

Originally published name:

Fatemeh Rahnemaie

Correct name:

Fatemeh Alsadat Rahnemaei

The original article has been corrected.

## References

[1] Simbar (2020). Is body image a predictor of women’s depression and anxiety in postmenopausal women?. BMC Psychiatry.

